# Tumor Reduction in Multiple Myeloma: New Concepts for New Therapeutics

**DOI:** 10.3389/fonc.2021.800309

**Published:** 2022-01-14

**Authors:** Rafael Alonso, Juan José Lahuerta

**Affiliations:** ^1^ Hematology Department, Hospital Universitario 12 de Octubre, CIBERONC CB16/12/00369, Madrid, Spain; ^2^ Instituto de Investigación Sanitaria, Hospital Universitario 12 de Octubre (imas12) CIBERONC CB16/12/00369, Madrid, Spain

**Keywords:** multiple myeloma, minimal residual disease, response criteria, complete response, NGS, NGF, MRD

## Abstract

The development of new resources for a more accurate diagnosis and response assessment in multiple myeloma has been a long process for decades, mainly since the middle of the 20th century. During this time, the succession of technical advances has run parallel to the better knowledge of disease biology and the availability of novel therapeutic strategies. The cornerstone of standardized criteria to uniformly evaluate the disease response in myeloma dates back to the 1990s when the key role of complete remission was established. Since then, different updates have been implemented according to available scientific evidences not always without certain controversies. The progressive improvements in survival results of myeloma patients and the growing quality of responses due to the novel therapies have led to the need of developing new tools for better monitoring of tumor burden. In this way, the concept of minimal residual disease and its key value based on the prognostic significance and the clinical relevance has been consolidated during the last years, overcoming the value of conventional response criteria or classical adverse prognosis markers. Nevertheless, its precise role in the clinical management of myeloma patients to detect early treatment failure and trigger early rescue strategies is still pending to be defined. In this review, we revisit the major milestones in the understanding of tumor reduction in multiple myeloma until the most recent imaging techniques or liquid biopsy approaches, including a critical view of conventional response criteria, whose backbone has remained unchanged during the last 20 years.

## Introduction

Multiple myeloma (MM) is a plasma cell neoplasm that represents the second most frequent hematologic malignancy. The natural history of MM is characterized by a succession of relapses interspersed with periods of remission of progressively shorter duration (1, 2), usually considered as an incurable disease for most patients. But, on the other hand, historical series have shown a significant improvement in survival outcomes since the last decades of the 20th century (3, 4) due to the introduction of novel drugs and combinations, the optimization of supportive treatment, a better knowledge of disease biology, and the implementation of new techniques for diagnosis and monitoring of MM. Simultaneously, the achievement of progressively deeper responses has been possible, and our understanding of tumor reduction in MM continues to expand. Nevertheless, some of the criteria for response assessment currently in force have remained mostly unchanged since they were established years or decades ago.

## The Control of Multiple Myeloma Through Monoclonal Immunoglobulins: A Historical Perspective

The first reports describing “Kahler’s disease” in the mid-19th century were focused on the clinical features and necropsy findings (5, 6). The presence of a monoclonal spike in the electrophoresis (EP) of serum proteins was not described until the late 1930s, but it was not until 1950 when EP and immunofixation (IF) were included as a tool for MM diagnosis in clinical practice. Moreover, although the first descriptions of urine Bence–Jones proteins date from the 19th century, they were only identified as the light chains of monoclonal immunoglobulins by Nobel Prize winner Gerald Edelman in 1963 (7).

The preliminary attempts to treat MM using experimental compounds with anticancer properties during the 1950s were unable to prolong the overall survival (OS) of MM patients (8, 9). Nevertheless, in 1962, melphalan demonstrated a significant cytotoxic effect on MM cells, which was enhanced through the combination with prednisone, achieving OS results longer than 3 years in MM patients (8, 10). The employment of monoclonal immunoglobulin in serum or urine to track tumor burden in those clinical studies led to the identification of a correlation between M spike reduction and improved OS outcomes (11). Nevertheless, none of the various combinations of chemotherapeutic agents explored during the 1960s, 1970s, and 1980s was able to overcome the results previously obtained with melphalan–prednisone (12–14).

The discovery of the powerful antitumor effect of high-dose chemotherapy followed by an autologous stem cell transplantation (ASCT) was a major milestone in the history of MM. This successful approach, originally led by the Royal Marsden Hospital (15) and the Arkansas group (16), resulted in the introduction of the concept of complete response (CR) by the end of the 1980s, which was then defined as the absence of M spikes in EP (17, 18). This notion of CR became more widespread during the 1990s, and it was even refined by the Arkansas group, who introduced the IF to define CR (19, 20). All these evidences supported the key role of ASCT in MM revealing for the first time a connection between depth and duration of response. A definitive phase III clinical trial published in 1996 by the Intergroupe Francophone du Myélome (IFM) showed that high-dose melphalan plus ASCT was superior to conventional chemotherapy, achieving higher CR rates assessed by EP and a significant benefit both in progression-free survival (PFS) and in OS (21).

## High-Dose Therapy and Autologous Stem Cell Transplantation: First Consensus Criteria

In 1998, Bladé *et al.* presented a proposal to define the response and progression criteria for MM in the Myeloma Subcommittee of the European Society for Bone and Marrow Transplant (EBMT) (22). This initiative was debated and agreed with other cooperative groups to include the results obtained from clinical trials with high-dose melphalan plus ASCT (23–25), and novel definitions replaced the consensus criteria stated during the polychemotherapy era when normalization of bone marrow plasmacytosis or loss of monoclonal bands in IF was never considered (26). The definition of CR reached at the time was later assumed by the International Myeloma Working Group (IMWG), and it still remains in force with few changes (27).

One meta-analysis gathering almost 5,000 patients with MM treated with high-dose melphalan and ASCT (28) confirmed the connection between CR and long-term prognostic improvement. Ten years later, a second meta-analysis (29) supported the value of CR in the present times. Nevertheless, the prognosis of MM patients who currently achieve a CR by employing new-generation drugs is strikingly better than that of those treated in earlier periods. Thus, this point suggests that certain differences may exist inside the CR category.

Intermediate responses between CR and partial response (PR), which were not considered in the EBMT classification, then appeared as new categories. They included the “near CR” (nCR), which was equivalent to CR by EP and the “very good partial response” (VGPR), advocated by the IFM and defined as a reduction of M spike at between 90% and 99% in EP. The IFM99-02/03/04 clinical trials found similar PFS/OS profiles in patients with CR and VGPR (30, 31). This fact promoted that VGPR became widespread, and it was eventually incorporated into the IMWG criteria in 2006 (32).

## Controversies on the Clinical Meaning of Complete Response in Multiple Myeloma

The stratification of disease response and its correlation with prognosis represented unquestionable progress, but the transfer to clinical practice was not without controversies, such as the denialism regarding the opportunity to consider CR and PFS extension as primary therapeutic endpoints. This debate, today surpassed, had a great impact involving relevant critics with this approach who argued toxicity reasons (33).

On the other hand, the incorporation of IF into clinical research was not homogeneous. The results from IFM studies reporting similar PFS/OS outcomes for patients in VGPR and in CR created confusion about the exact role of CR and led to the overestimation of the value of VGPR. Nevertheless, the achievement of CR was only based on a negative EP since IF was not employed in these studies; thus, both categories were almost identical (31).

The IMWG criteria from 2006 introduced the new category of stringent CR (sCR), which included the normalization of serum-free light chain ratio and absence of clonal cells in bone marrow biopsy by immunochemistry in addition to the requirements for CR (27). New controversies emerged regarding this novel category since the favorable results obtained in some studies using these criteria (34) were not confirmed by other authors who questioned the usefulness of sCR due to the low sensitivity and specificity of fluorescence and the absence of differences in survival when it is compared with CR (35–37).

Since 1998, the definition of CR according to the IMWG criteria has invariably required the absence of monoclonal paraprotein in serum and urine by IF. In 2004, an External Committee published a proposal for uniform assessment and reporting of responses in clinical trials under the supervision of Independent Committees. This outlook recommended 2 consecutive evaluations with negative IF in both serum and urine to confirm CR, classifying the response as VGPR when serum IF was negative but no urine IF was available (38). This downgrading of CR to VGPR in the absence of urine assessments led to an inappropriate reduction of CR rate in some clinical trials (39, 40), a relevant end-point for efficacy. A recent sub-analysis of phase III GEM2012menos65 clinical trial (41) queries this point by showing a 0% urine IF-positive rate in 107 patients with serum M-protein at diagnosis who became serum IF-negative after treatment. Meanwhile, in 161 patients with both serum and urine M-protein at diagnosis who became serum IF-negative after treatment, only 1.8% was urine IF-positive.

In addition, when prognosis according to the depth of response was evaluated in 449 patients from the GEM2012menos65 trial, the conventional response criteria showed a limited value for prognosis, especially in patients already installed in the maintenance phase, and globally in patients with persistent measurable residual disease (MRD), a concept very similar to minimal residual disease (42). There were no differences in PFS or OS for patients with MRD-positive status irrespective of whether they have achieved PR, VGPR, CR, or sCR (PFS, p > 0.08; OS, p > 0.2); and plasma cell count in bone marrow and free light chains ratio did not have prognostic impact in patients with negative IF (43).

## Novel Perspectives in Tumor Burden Assessment: The Minimal Residual Disease or the Measurable Residual Disease

Despite the value of CR in clinical practice, the prognosis associated with CR is heterogeneous. Overall, those therapeutic combinations with higher CR rates are associated with longer PFS in comparison with CRs obtained with other therapies with lower efficacy (44). The variability in the length of CR seems consistent with the results of the aforementioned meta-analysis (29), which showed a better performance of CRs achieved with novel drugs in comparison with those obtained with chemotherapy.

Mass spectrometry (45) or the detection of persistent tumor cells in bone marrow or peripheral blood with high-sensitivity methods based on molecular (46) or immunophenotypic (47) techniques represent the answer to this need. Additionally, high-resolution imaging techniques increase the potential to identify the underlying disease (48). Multiparametric flow cytometry (MFC) has become the most employed technique to assess MRD due to its prompt availability and the elaboration of a comprehensive standard as EuroFlow (49).

Even with 4-color immunofluorescence techniques and sensitivity of 10^−4^, MFC in bone marrow confirmed its clinical relevance since very early approaches. The first analyses of the GEM2000 trial, in the era of chemotherapy and ASCT, demonstrated substantial differences in prognosis between patients who achieved MRD-negative status at day 100 after ASCT and those who maintain MRD-positive status (median PFS 71 vs. 37 months, p < 0.001; median OS not achieved vs. 89 months, p = 0.002). Even more, in a combined analysis of GEM2000 and GEM05menos65 clinical trials, patients with MRD-negative or MRD-positive status showed similar prognosis in each subgroup regardless of the induction scheme with chemotherapy or novel combinations including proteasome inhibitors and immunomodulatory drugs (GEM data not published).

More recently, an integrated evaluation of 3 phase III GEM/PETHEMA clinical trials including new drugs confirmed that the MRD-negative rates (sensitivity 10^−4^) after different induction regimes anticipate longer PFS, reinforcing the key value of MRD in the efficacy assessment of new treatments. In this analysis, the achievement of CR without MRD-negative did not improve PFS/OS outcomes in comparison with patients in PR or VGPR. The benefit associated with MRD negativity was consistent in all subgroups analyzed including patients with high-risk cytogenetics (50). Further studies including patients treated with different combinations and similar sensitivity thresholds have validated these results (51).

## Consolidation of Measurable Residual Disease in the Clinical Management of Multiple Myeloma

### Flow Cytometry

Second-generation MFC, which achieves a sensitivity of 10^−5^, increases the power of the MRD to discriminate patients with different prognoses over the aforementioned techniques. In phase III GEM2010 clinical trial including ≥65-year-old patients with newly diagnosed MM (NDMM), the achievement of MRD-negative by second-generation MFC strikingly manage to overcome the adverse prognosis associated with high-risk cytogenetics in comparison with standard-risk patients (52, 53). Overall, a sequential improvement in PFS/OS outcomes was observed per tumor burden logarithmic depletion (54). This point justifies the exploration of more sensitive techniques to detect the remaining tumor burden.

The EuroFlow standard (49) implements a novel flow cytometry approach (next-generation flow [NGF]) to identify MRD with a deeper sensitivity of 10^−6^. Second-generation MFC and NGF have demonstrated a good correlation when tumor burden is relatively high (PR, VGPR, CR), but approximately 25% of patients with MRD-negative status by conventional MFC became MRD-positive when they were assessed by NGF. In the recent intention-to-treat analysis of ASCT-eligible patients with NDMM included in the GEM2012menos65 trial (55), those patients with MRD-positive status by NGF showed an 82% reduction in the risk of progression or death (hazard ratio 0.18, p < 0.001) in comparison with MRD-negative patients. These results support the role of the achievement of MRD-negative status to overcome the penalty associated with risk factors including high-risk cytogenetics.

### Molecular Techniques

Clonal rearrangements of immunoglobulin genes detected by fluorescence PCR or the more complex and sensitive allele-specific oligonucleotide PCR (ASO-PCR) have shown to be useful to measure MRD and discriminate groups of patients with different prognoses at the frontline (56, 57).

In the past few years, deep sequencing (next-generation sequencing [NGS]) with a sensitivity of 10^−5^/10^−6^, or even deeper than 10^−6^, has been the molecular technique of choice to assess MRD in MM in many studies (46, 58, 59). NGF and NGS have demonstrated a high degree of agreement when they are compared at the same level of sensitivity (46, 60).

### The Settlement of Measurable Residual Disease in Clinical Practice

The IMWG criteria for response assessment from 2016 (27) prompted the new category of CR with MRD-negative status indistinctly defined by LymphoSIGHT (or any alternative validated NGS method) or by MFC according to EuroFlow standard, achieving at least a sensitivity of 10^−5^. This classification included a novel category of “sustained MRD-negative” for patients with MRD negativity in the marrow and by imaging confirmed minimum in 2 consecutive evaluations at 1 year apart.

Recent meta-analyses confirmed the key improvement both in PFS and in OS for patients who achieve MRD-negative status, with or without CR (61, 62). Remarkably, the survival benefit associated with MRD-negative status was observed in ASCT-eligible and non-eligible NDMM patients, but also in patients with relapsed/refractory MM (RRMM), and even overcoming the adverse prognosis of high-risk cytogenetics. Further studies have supported these evidences (63, 64).

Therefore, the obtainment of an MRD-negative status represents a valid surrogate marker for PFS (and likely also for OS) in almost every scenario in MM. A growing number of opinions are claiming the recognition of MRD negativity as a primary end-point for efficacy in clinical trials (65–68). Additionally, many studies have confirmed the prognostic power of MRD kinetics based on the evidences obtained during the maintenance phase of treatment at the frontline (69–71).

The first results from clinical trials where treatment is modulated according to MRD results are beginning to become available (72, 73). Furthermore, novel studies including ultra-early salvage therapy are now being designed, supported by the hypothesis that when tumor burden is low, MRD kinetics would be especially useful for the early detection of the initial signs of therapeutic failure (74). This would enable the taking of measures to manage early treatment and to abort the emerging progression.

### When a Single Bone Marrow Is Not Enough: The Role of Imaging Techniques

Sometimes, a single bone marrow aspirate or biopsy may be insufficient to obtain the full picture of the extent of MM due to the heterogeneous bone marrow infiltration or the presence of extramedullary disease. Today, the conventional skeletal X-ray survey tends to be replaced by alternative imaging techniques since it requires 30%–50% of trabecular bone destruction to detect bone damage, and it is not useful to discriminate between residual and active lytic lesions (75).

Positive lesions in PET-CT scans have shown adverse prognostic value both at diagnosis and at relapse (48, 76). Additionally, the intake suppression of known lesions before or after ASCT has a favorable impact on PFS and OS (77, 78). In fact, a new category of imaging plus MRD-negative status (which requires a response by PET-CT in addition to MRD negativity) was recognized in the last consensus of the IMWG (27), underscoring the complementarity between both approaches (79, 80). Different proposals for standardization have been implemented in recent years, and they are pending validation (81, 82).

Moreover, MRI is the gold-standard imaging technique to assess bone marrow. This fact is important since the bone marrow infiltration by MM cells may be heterogeneous, and the identification of focal lesions in bone marrow by MRI has proven to have a prognostic value (83, 84).

## Towards a Comprehensive Evaluation of Tumor Burden: The Future

New techniques of mass spectrometry (MS) enable the identification of monoclonal proteins with a deeper limit of sensitivity in comparison with EP and IF (85), leading to an increased power to discriminate populations with different survival outcomes. It is still pending validation in large prospective studies, but preliminary data suggest that MS may represent a less invasive alternative than MRD evaluations in bone marrow by NGS or MFC (45, 86).

The detection of circulating tumor cells (CTC) in peripheral blood may be representative of the residual tumor burden, being a minimally invasive approach in the setting of precision medicine (87). In fact, the identification of CTC is associated with prognostic significance, and it has an impact on survival results (88, 89). Recent and innovative techniques such as the detection of circulating microRNA, cell-free circulating tumor DNA, or extracellular vesicles released from MM cells may be useful in the clinical monitoring, the evaluation of tumor heterogeneity and clonal evolution, or the identification of therapeutic targets (90).

Other advances are also being implemented in the setting of imaging techniques. Whole-body diffusion-weighted MRI (WB-DWI) maintains the advantages of MRI to identify small lesions in bone marrow at diagnosis, but it also allows the quantification of residual disease through the apparent diffusion coefficient without the need for intravenous contrast and shows a good correlation with bone marrow infiltration, thus being superior to conventional MRI (91, 92). Some preliminary studies have confirmed a benefit in PFS linked to the achievement of WB-DWI negativity after ASCT (93, 94), additionally being a technique without ionizing radiation, which may overcome some of the limitations of PET-CT (95).

## Discussion: Winds of Change for Response Criteria in Multiple Myeloma

CR in MM patients is linked with a clear improvement in survival outcomes, also in the age of novel agents, but it is losing consistency in the long term. The possibility of detecting and quantifying deeper thresholds of tumor burden has begun to blur the usefulness of conventional response criteria, undermining the role of traditional response categories.

For most of the MM patients, especially at the frontline or at first relapses, the achievement of MRD-negative status in bone marrow represents a potential surrogate marker for PFS, which is able to overcome the adverse prognosis of classical factors as the high-risk cytogenetics. In recent years, MRD negativity has been progressively consolidated as a primary end-point to evaluate the efficacy of new therapies in clinical trials.

Bone marrow techniques for MRD assessment may be insufficient to accurately estimate the residual tumor burden. To this end, new imaging techniques (PET-CT or WB-DWI) or serum approaches (MS and liquid biopsy) have been implemented, showing a potential complementarity with current MRD studies (MFC or NGS).

Breakthrough therapeutic agents and combinations, optimized supportive therapies, and new diagnostic techniques are improving the management of MM patients and their survival outcomes. All these changes have brought scenarios and questions that require new answers: when and how to measure MRD. Should we advocate for the early detection of treatment failure and consequently promote early rescue interventions? Is now the time for clinical decision making based on MRD results?

## Data Availability Statement

The original contributions presented in the study are included in the article/supplementary material. Further inquiries can be directed to the corresponding author.

## Author Contributions

Writing, original draft preparation, review, and editing were accomplished by JL and RA. Both authors have read and agreed to the published version of the manuscript.

## Funding

This work was funded by the Programa Español de Tratamientos en Hematología (PETHEMA) Foundation.

## Conflict of Interest

The authors declare that the research was conducted in the absence of any commercial or financial relationships that could be construed as a potential conflict of interest.

## Publisher’s Note

All claims expressed in this article are solely those of the authors and do not necessarily represent those of their affiliated organizations, or those of the publisher, the editors and the reviewers. Any product that may be evaluated in this article, or claim that may be made by its manufacturer, is not guaranteed or endorsed by the publisher.
